# Design of a Phase IIb, randomized, controlled, double-blind, multi-centre study to evaluate the efficacy, safety, and immunogenicity of the GMZ2 candidate malaria vaccine in Ugandan, Ghanaian, Burkinabe and Gabonese children aged 12-60 months

**DOI:** 10.1186/1475-2875-9-S2-P26

**Published:** 2010-10-20

**Authors:** Ramadhani A Noor, Michael Thiesen, Benjamin Mordmuller, Paul Milligan, Frank Atuguba, Sodiomon Sirima, Saadou Issifou, Kalifa Bojang, Fred Kironde, Alfred Tiono, Brenda Okech, Roma Chilengi, Ateba Ngoa, Kaddu Mukasa, Soren Jepsen

**Affiliations:** 1 African Malaria Network Trust (AMANET), Dar Es Salaam, Tanzania, United Republic of; 2 Statens Serum Institut (SSI) Denmark, Copenhagen, Denmark; 3 Universitätsklinikum Tübingen (UKT), Tübingen, Germany; 4 London School of Hygiene and Tropical Medicine (LSHTM), London, UK; 5 Navrongo Health Research Centre (NHRC), Navrongo, Ghana; 6 CNRFP, Burkina Faso, Ouagadougou, Burkina Faso; 7 Medical Resaerch Unit-Albert Schweitzer Hospital, Lambarene, Gabon; 8 Medical Research Council (UK)The Gambia, Fajara/Banjul, Gambia; 9 Makerere University, Kampala, Uganda; 10 KEMRI-Wellcome Trust Research Programme, Mombasa, Kenya

## 

The malaria vaccine candidate GMZ2 is a recombinant fusion protein of Plasmodium falciparum Glutamate Rich Protein and Merozoite Surface Protein 3, adjuvanted with aluminium hydroxide. Trials in malaria naive adults in Germany and in Gabon in adults exposed to malaria and subsequently in children 1-5yrs showed that three doses of 100ug GMZ2 are well tolerated and immunogenic. The aim of this phase 2b trial is to determine whether the GZ2 vaccine can protect against clinical attacks of malaria in children aged 1-5yrs, to obtain more extensive data on safety, tolerability and immunogenicity, and to determine if the vaccine can prevent anaemia and reduce the parasite density and gametocyte carriage.

The primary endpoint is the incidence of clinical malaria defined as fever or history of fever in the previous 24 hours with parasite density of 5000 asexual parasites per ul or more, detected by passive surveillance over a 6 month period from the third vaccination. All malaria episodes will be included in the calculation of vaccine efficacy. This endpoint is chosen in order to have the optimum power to detect a protective effect; efficacy will also be calculated using a range of different parasite density cutoff values. Children will be followed for a total of 22 months from the third vaccination. Immune responses to the vaccine antigens GMZ2, GLURP and MSP3 will be assessed by measuring antigen specific IgG by ELISA, and antigen specific memory B-cell responses by ELISPOT. Functionality of the immune response will be assessed by growth inhibition of P. falciparum in the presence or absence of monocytes. Cell mediated immunogenicity will be assessed by cytokine profiling and intracellular cell staining following stimulation with the vaccine antigen, and the quality of the antibody response by type and subclass-specific ELISA.

The planning of the trial was complicated by the need to take account of the changing epidemiology of malaria. Baseline studies were conducted in five potential trial sites to determine the current incidence of malaria; in each site two cohorts of children were followed to estimate the incidence of malaria by active and passive surveillance and by passive surveillance alone. Four sites with higher malaria incidence were chosen for recruitment. A total sample size of 1840 will be enrolled in each of the four sites in 2010/11.

